# Supplementary material for: Patterns of Intron Gain and Loss in Fungi
Source: PLoS Biol. 2004 Nov 30;2(12):e422. doi: 10.1371/journal.pbio.0020422 (PMC532390; doi:10.1371/journal.pbio.0020422)
Supplement: Table S1 — Also available at http://genes.mit.edu/NielsenEtAl/. (4.3 MB ZIP). [file pbio.0020422.st001.zip › NielsenEtAl/html/819.html]

AN8857.1.NCU00417.1.MG06309.1.FG06935.1


```
 CLUSTAL W (1.82) Multiple Sequence Alignments - Introns Inserted


Sequence 1: MG06309.1	148 aa
Sequence 2: FG06935.1	165 aa
Sequence 3: NCU00417.1	148 aa
Sequence 4: AN8857.1	174 aa
Alignment Length: 191 aa
Number Identitical Residues: 93 aa
Alignment Score (without introns) 4564


MG06309.1 	MPAIRHASKRKPPPEGFSDIEDDLLIFANKMKDAQNTPTDNIPKHKAQWPIFQIAHQR~S
NCU00417.1	MPAIRPASKRKPPPEGFADIEEDLLIFANKMKDAQNAPTDNIPKHQAKWPIFQISHQR~S
FG06935.1 	MPAIRHSSKRKPPPDGFEDIENDLLIFANKMKDAQNKPPPSGPRHQAQWEIFQISHQR~S
AN8857.1  	MPPIRTSRNRKPPPAGFDDIEDTLLEFSNKMKDAENAPHEGKKKHEVLWPIFQITHQR1S
          	**.** : :***** ** ***: ** *:******:* *  .  :*:. * ****:*** *

MG06309.1 	RYVYELYYEKEAISKQLYDWLLKNGYADPMLIAKWKKQGYEK0-----------------
NCU00417.1	RYIYELYYEKEAISKQLYEWLLKNGYADAMLIAKWKKNGYEK~-----------------
FG06935.1 	RYVYELYYEKEAISKKLYDWLLKNGYADAMLIAKWKKQGYEK~VCLFNHHPFCANTNISQ
AN8857.1  	RYIYDLYYQKEAISKQLYEWLLKNGYADANLIAKWKKQGYEK0-----------------
          	**:*:***:******:**:*********. *******:****                  

MG06309.1 	LCCLRCVQTKETNFSSTCICRVPKAQLKEERDIE~------------CVNCGCHGCASSD
NCU00417.1	LCCLRCIQTKETNFNSTCICRVPKAELKEGQDVQ~------------CVSCGCRGCATGD
FG06935.1 	LCCLRCIQTKETNFNSTCICRVPRAEMKGDEDIE~------------CVSCGCRGCASSD
AN8857.1  	LCCLRCIQTKETNFNATCICRVPKAQLKEDQIIH2NSVGPKAAGQQPCCSPGLRRSQSRR
          	******:*******.:*******:*::*  . :. .: ...::....* . * : . :  

MG06309.1 	-----------~---
NCU00417.1	-----------~---
FG06935.1 	-----------~---
AN8857.1  	RCGVPIIPIFH0ISS
          	 .. .  .     ::
```
